# A case of hepatocellular adenoma with pedunculated development and difficulty in diagnosis

**DOI:** 10.20407/fmj.2019-005

**Published:** 2019-11-02

**Authors:** Norihiko Kawabe, Takahiko Higashiguchi, Hironobu Yasuoka, Toki Kawai, Kenshiro Kamio, Takayuki Ochi, Chihiro Hayashi, Masahiro Shimura, Shinpei Furuta, Satoshi Arakawa, Yuka Kondo, Yukio Asano, Hidetoshi Nagata, Masahiro Ito, Akihiko Horiguchi, Zenichi Morise

**Affiliations:** 1 Department of Gastroenterological Surgery, Fujita Health University Bantane Hospital, Nagoya, Aichi, Japan; 2 Department of Surgery, Fujita Health University, School of Medicine, Toyoake, Aichi, Japan

**Keywords:** Hepatocellular adenoma, Benign liver tumor, Pedunculated development

## Abstract

Hepatocellular adenoma (HCA) is a benign hepatocyte-derived epithelial tumor. HCA is associated with oral contraceptive use among Caucasian populations. We report a case of hepatocellular adenoma with a pedunculated protuberance and high protein induced by vitamin K absence or antagonist-II (PIVKA-II) levels, which made diagnosis challenging. The patient was a 22-year-old woman. In a medical check-up, a high γ-GTP level was detected and a 115-mm solid mass was found in her lower abdomen via abdominal ultrasonography. A blood test showed a high PIVKA-II level. Abdominal CT showed a tumor in the lower abdomen. Contrast-enhanced CT showed a blood vessel thought to be the left hepatic artery connecting to the mass, and a blood vessel thought to be the left hepatic vein returning from the mass to the inferior vena cava. In EOB-MRI, uneven enhancement was observed after contrast imaging, but washout in the equilibrium phase was unclear. Parenchymal hepatocyte phases showed a pale, non-uniform, high signal. These findings indicated that the tumor was derived from the left lobe of the liver and was suggestive of HCC. Surgical resection was then performed. A pathological examination led to a diagnosis of HCA, corresponding to unclassified HCA. The WHO classification of tumors of the digestive system based on an immunohistological examination includes HNF1α-inactivated HCA, β-catenin-activated HCA, inflammatory HCA, and unclassified HCA. In summary, our patient had a large HCA with pedunculated protrusion into the extrahepatic pelvic cavity. This case was challenging to diagnose because of abnormally high PIVKA-II levels, and it was resected laparoscopically.

## Introduction

Hepatocellular adenoma (HCA) is a benign hypervascular tumor that is composed of cells resembling normal hepatocytes.^1^ The incidence of HCA in young women is high and associated with oral contraceptive use in the USA and Europe. However, the incidence of HCA in Asian women is not as high as that in other countries, and this is thought to be due to differences in oral contraceptive use rates among countries.^1,2^ In 2010, the World Health Organization (WHO) classification of tumors of the digestive system was revised and included four genetic phenotypes as follows: hepatocyte nuclear factor 1α (HNF1α)-inactivated HCA(H-HCA), β-catenin-activated HCA(b-HCA), inflammatory HCA(I-HCA), and unclassified HCA(u-HCA), each of which has characteristic clinical imaging, and pathology findings.^3^ We report here a case of a large HCA with pedunculated protrusion into the extrahepatic pelvic cavity. This case was challenging to diagnose because of an abnormally high protein induced by vitamin K absence or antagonist-II (PIVKA-II) level. The tumor was resected laparoscopically.

## Case

The patient was a 22-year-old woman. A high gamma‐glutamyl transpeptidase (γ-GTP) level was noted during a health check-up and she visited a local doctor 11 months later.

*Examination findings*: Abdominal ultrasonography showed a solid mass of 115 mm in diameter in the abdominal cavity. Although there were signs of pressure on the surrounding organs, continuity with these organs could not be confirmed. However, a tumor originating from the ovaries was suspected and she was referred to our hospital on the following day for further tests.

At her first visit, she had good appetite and there was no weight loss or notable digestive symptoms. There was no noteworthy medical history and no history of oral contraceptive use. Her height was 163 cm, weight was 49 kg, body temperature was 36.2 C, and eyeballs and palpebral conjunctiva were normal. Blood pressure, pulse, and respiratory sounds were normal. No heart noise was heard and no edema on the face or limbs was present. On abdominal palpation, a fist-sized elastic, slightly hard, smooth mass was found under the umbilical cord. General blood biochemical tests showed no increase in any parameter other than the γ-GTP level. Blood tumor marker tests showed a high PIVKA-II level (Table 1).

**Figure 1:**
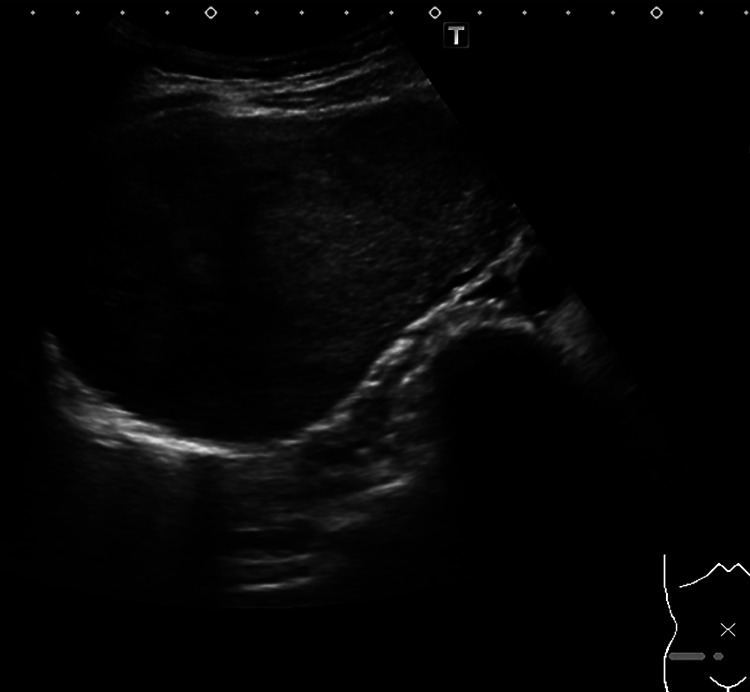
Abdominal ultrasound A multi-compartmented mass with 126×72-mm lining was observed in the right lower abdomen. The inside of the mass had a slightly high echo and there were some uneven parts.

Abdominal ultrasonography showed a multiloculated mass with a size of 126×72 mm in the right lower abdomen, a slightly hyperechoic interior with non-uniform areas, and no obvious vasculature. Continuity with surrounding organs could not be confirmed (Figure 1).

**Figure 2:**
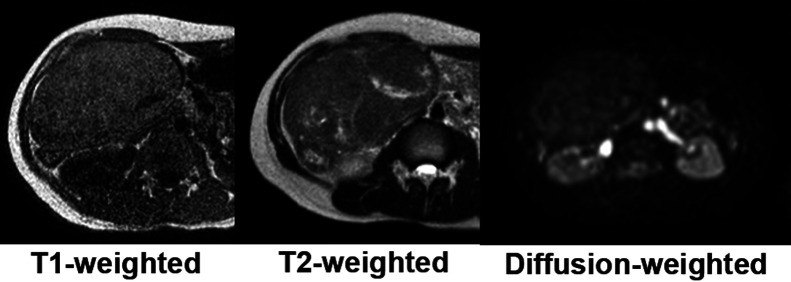
MRI The tumor was solid in the right upper abdomen, it was separated from the uterus and ovaries, and no obvious abnormalities were found in the pelvis. A T1-weighted image shows a light high signal. A T2-weighted image shows an iso-signal with a non-uniform high signal internally. A diffusion-weighted image shows a low signal.

Plain abdominal magnetic resonance imaging (MRI) showed a solid tumor in the right upper abdomen. Apart from the uterus and ovaries, there were no other obvious abnormalities in the pelvis. T1-weighted imaging showed a light high signal. T2-weighted imaging showed an iso-signal with a non-uniform high signal internally and diffusion-weighted imaging had a low signal (Figure 2). Although the relationship with the intestinal tract was not clear, the possibility of a gastrointestinal stromal tumor was also considered.

**Figure 3:**
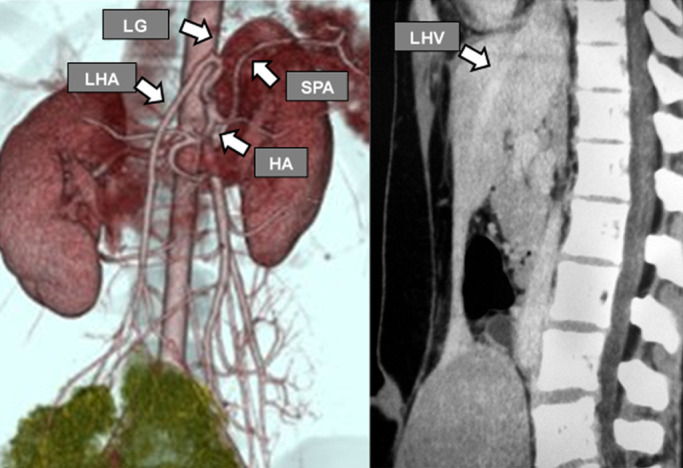
Dynamic CT angiogram The tumor was infused with continuous arteries from the left hepatic artery, and the vein returned to the inferior vena cava via the left hepatic vein. LG: left gastric artery; HA: hepatic artery; LHA: left hepatic artery; SPA: splenic artery; LHV: left hepatic vein.

**Figure 4:**
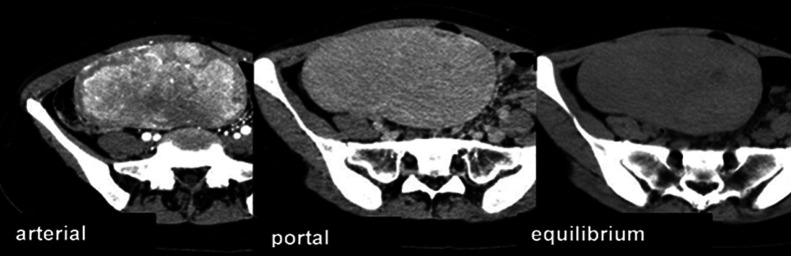
Dynamic CT The tumor was located in the lower right abdomen, and was outlined with dense staining in the arterial phase and low absorption in the equilibrium phase.

Abdominal contrast-enhanced computed tomography (CT) showed a mass of 130×145×70 mm in the lower right abdomen, and this was depicted in the lower pelvis from the time of simple MRI. The tumor was infused by an artery continuous with the left hepatic artery, and a vein was seen returning to the inferior vena cava via the left hepatic vein (Figure 3). The normal lateral segments of the left hepatic lobe were small and appeared to be continuous with the tumor. Therefore, a tumor in the intrathecal region or the lateral segments of the left hepatic lobe was suspected. In the early phase of contrast enhancement, non-homogeneous enhancement was observed. In the equilibrium phase, non-homogeneous light-dark staining was prolonged. Mild washout was observed compared with the early phase, and hepatocellular carcinoma (HCC) or HCA in the left hepatic lobe was suspected (Figure 4).

**Figure 5:**
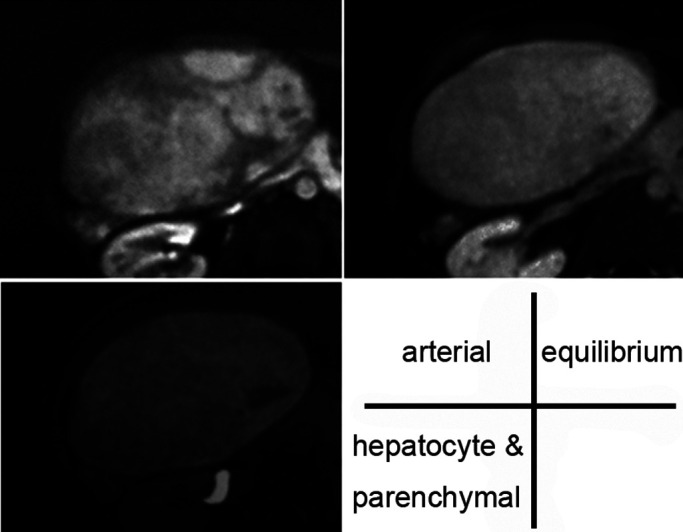
Contrast MRI With a non-uniform enhancement effect after contrast in EOB, washout in the equilibrium phases was not clear. In the hepatocyte and parenchymal phases, it showed a pale, non-uniform, high signal.

Abdominal contrast-enhanced MRI (EOB) showed an uneven enhancement effect after contrast. In the equilibrium phase, washout was not clear and the hepatocyte parenchymal phase showed a pale, non-uniform, high signal (Figure 5). HCC was also suspected, but washout was poor, and no high signal was present in a diffusion-weighted image of plain MRI.

On the basis of the above-mentioned results, because the tumor was located in the left lobe of the liver, and because HCC could not be ruled out because of early staining after contrast and the high tumor marker level, surgery was performed 2 months after the first examination.

*Surgical findings*: Port insertion with an umbilical longitudinal incision open method was performed. A 12-mmφ port in the left flank and a 5-mmφ port under the spinous process were inserted. A pedunculated tumor continuous with the left lobe of the liver was confirmed.

**Figure 6:**
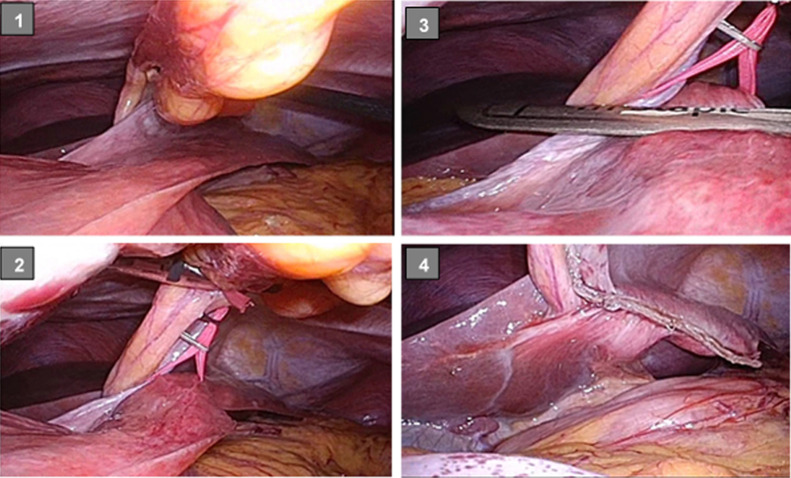
Surgical findings A pedunculated continuous tumor extending from the left lobe of the liver to the side of the leg was identified. Tape was applied to the stem to lift the tumor up. Using a laparoscopic linear stapler (1 mm) (white), the tumor was separated into two steps. No bleeding or bile leakage was observed in the section after separation.

The neck connecting the tumor and the lateral liver area was approximately 4 cm wide, approximately 5 cm long, and approximately 5 mm thick at the largest part. After raising the neck with vascular tape, resection was performed using a laparoscopic linear stapler twice. A skin incision of approximately 10 cm in length was made in the upper pubic bone and the tumor was removed. The surgical duration was 2 hours and 35 minutes, and the amount of bleeding was 30 g (Figure 6).

**Figure 7:**
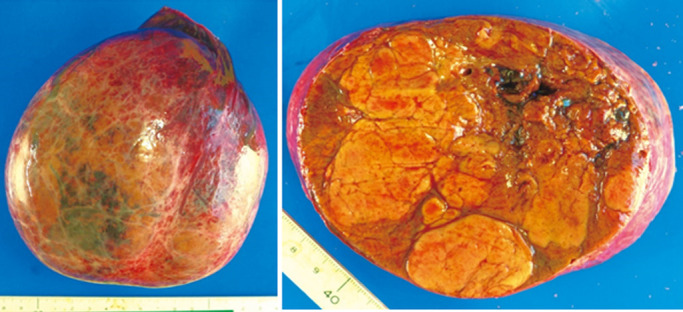
Resected specimen The tumor was 15×13×9.5 cm in size, it was nodular, there was no dilatational growth, and it showed capsule formation, but no capsule invasion. Partition wall formation was observed inside the tumor. There was no infiltration into the serous membrane.

*Specimen findings:* A nodular and distended tumor of 150×130×95 mm with capsule formation, but no capsular invasion, was observed. Formation of partitions was observed inside the tumor. There was no infiltration into the serous membrane, portal vein, hepatic vein, hepatic artery, or bile duct, and no lymph node metastasis (Figure 7).

**Figure 8:**
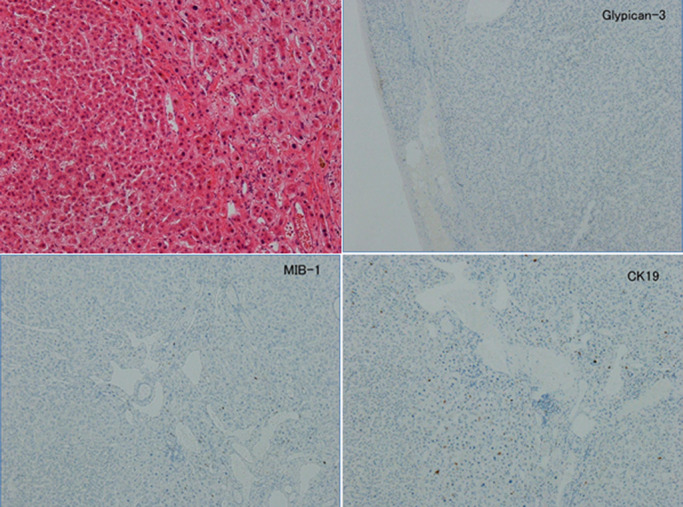
Pathological diagnosis Hematoxylin and eosin staining showed a large, nodular, hepatocellular tumor with relatively thin connective tissue lining. The tumor was negative for MIB-1, cytokeratin 19, and glypican-3, and had no clear image of intrahepatic hepatocellular infiltration. Therefore, the tumor was diagnosed as hepatocellular adenoma.

In histopathological analysis, hematoxylin and eosin staining showed a large, nodular, hepatocellular tumor with relatively thin connective tissue. The tumor cells formed hepatocyte cord-like structures and their structure was dysplastic. No production of bile was observed. The small amount of stroma involved contained vasodilation, but no bile ducts. An immunohistological examination showed that Mind Bomb(MIB)-1 was negative, cell proliferation was low, cytokeratin 19 was negative, and no bile duct components were present. The patient was negative for glypican-3 and no apparent signs of interstitial hepatic cell infiltration were found. She was diagnosed with HCA (Figure 8).

**Figure 9:**
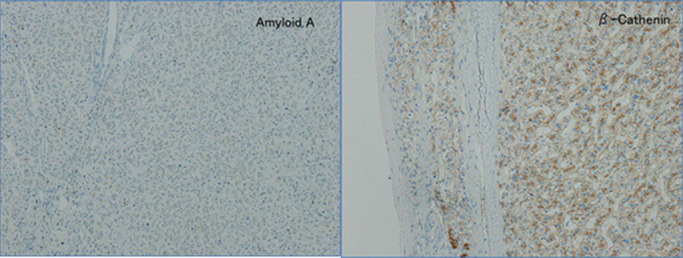
General pathological findings and immunohistochemical findings There was no diffuse steatosis. Amyloid A was negative, and although there was some vasodilation, cell infiltration was not observed. β-catenin was negative.

On the basis of the above-mentioned findings, among the four subtypes specified in the latest 2010 WHO classification of HCA, H-HCA was ruled out because of the absence of histological features, diffuse steatosis, or a history of oral contraceptive use. I-HCA, which is the most frequent subtype, was ruled out because the tumor was amyloid A-negative. b-HCA was ruled out because the tumor was β-catenin-negative. Therefore, she was diagnosed with u-HCA (Figure 9).

Postoperative course: The postoperative course was good, and the patient was discharged on the 7th day after surgery. The PIVKA-II level became normal 1 month after surgery and no recurrence was observed 4 years later.

## Discussion

HCA is a benign tumor derived from liver cells that occur in the normal liver and has an occurrence rate of three to four per 100,000 people in the USA and Europe. A total of 85% of cases of HCA occur in young women aged 20–40 years, and 80% to 90% of female patients are reported to have used oral contraceptives. The percentage of women diagnosed with HCA in Asian countries including Japan is approximately 50% to 60%, and this lower rate is thought to be due to the difference in oral contraceptive use between countries. An association of HCA with other anabolic hormones and glycogenosis has been reported.^2^ HCA may also be complicated by intrahepatic portal blood flow abnormalities, such as portal vein defects, obstruction, or portal vein shunt formation.^4^ The main reasons for discovery of HCA are pain, symptomatic hemorrhage, and liver dysfunction, and accidental detection in one third of the cases. In the present case, the background factors were relatively consistent with those of HCA, although there was no history of oral contraceptive use.

Distinguishing HCA from well-differentiated hepatocellular carcinoma and focalized nodular hyperplasia (FNH) may be challenging.^1^ FNH has no background factors found in HCA, such as use of oral contraceptives, alcohol consumption, obesity, or glycogenosis. In FNH, central scarring and abnormal blood vessels are features observed in images, and the hepatocellular phase of EOB-MRI shows a high signal of 90% or more.^5^ Histologically, glutamine synthetase–positive cells are distributed in a map-like form. Because there is no possibility of bleeding or canceration, if FNH can be diagnosed, there is no indication for surgical treatment. However, HCA is a neoplastic growth of hepatocytes with poor or almost no histopathology, and as a complication, intraperitoneal hemorrhage from this tumor can be fatal. Additionally, there are also reports of HCA leading to cancer, although the frequency is low.^6,7^

Upon differentiation from hepatocellular carcinoma in MRI using EOB, T1-weighted imaging of HCC shows that, as the degree of differentiation increases, a change from an uneven low signal to a high signal is observed. In T2-weighted imaging, a moderately high signal is seen, and in diffusion-weighted imaging, a high signal is seen. In the equilibrium phase, a low signal is observed, and in the hepatocyte phase, a low signal is observed. In the case of HCA, there are various characteristics depending on the subtype, and no specific findings have been reported.^5^ In the present case, washout was poor, and no high signal in a diffusion-weighted image was observed. However, the PIVKA-II level was high, and the possibility of preoperatively, well-differentiated hepatocellular carcinoma could not be ruled out. Preoperative diagnosis of HCA or well-differentiated hepatocellular carcinoma is considered an indication for surgery.

In the present case, formation of central scars and fibrous septa, which is characteristic of FNH, was not observed in preoperative imaging or postoperative pathological findings. Because there was also no ductular reaction or dilatation, our case was considered negative for FNH. Immunostaining was negative for cytokeratin 19, which is present in the biliary duct epithelium. Immunostaining also showed a low score for MIB-1, which is a monoclonal antibody recognizing Ki-67, a marker of proliferating cells in tumor tissue. Because the tumor was also negative for the biomarker glypican-3, well-differentiated hepatocellular carcinoma was excluded, and the patient was finally diagnosed with HCA.^8^

Possible mechanisms for PIVKA-II production in HCC include qualitative or quantitative abnormality of prothrombin precursors, altered activity of gamma-glutamyl carboxylase, and abnormality in the vitamin K cycle or a lack of vitamin K.^9^ Similar mechanisms for PIVKA-II production could occur in HCA. The possible consequences of hepatocellular dysfunction induced by neoplastic changes in HCA have been considered.^10^ Elevation of PIVKA-II levels is common in cases with malignant transformation, but high values may be found, even if there is no malignant change. Furthermore, no correlation of PIVKA-II levels with the WHO classification or clear features have been found.

According to the 2010 revision of the WHO classification of tumors of the digestive system, HCA was classified into the following four subtypes.^3^ H-HCA shows absence of liver fatty acid binding protein in immunostaining, and accounts for 35% to 40% of all HCAs. In H-HCA, most cases are women, and histologically, it is characterized by diffuse steatosis and may be single or multiple. Although β-catenin-activated HCA is infrequent, accounting for 10% to 15% of cases, the risk of cancer is high compared with other subtypes, and it is clinically important. Additionally, there are also many reported cases of β-catenin-activated HCA in men. Immunohistologically, glutamine synthetase is diffusely positive and β-catenin is positive in the nucleus; it often occurs singly. I-HCA is the most common subtype, accounting for 45% to 60% of cases. Inflammation-related proteins are found in I-HCA (e.g., tumors are positive for serum amyloid A or C-reactive protein) Histologically, focal or diffuse inflammation is seen in I-HCA, along with sinusoid dilatation, congestion, purpura, and bile duct hyperplasia. u-HCA has a frequency of 10% or less, has no immunohistological features, and none of the above-mentioned features are applicable.^1,2^ Although u-HCA accounts for approximately 10% of all HCA cases in Caucasian populations, its rate is approximately 30% in Japan, which is notable.^2^ As mentioned above, differences in oral contraceptive use among countries may be the cause of this different in rate.

In recent years, a new subgroup classification of HCA related to risk factors, clinical behavior, and histological features has been proposed (HCA molecular classification in 2017). Based on the results of gene analysis, HCA is classified as H-HCA, I-HCA, CTNNB1 mutation of exon 3 HCA (b^ex3^HCA), CTNNB1 mutation of exons 7 and 8 HCA (b^ex7,8^HCA), sonic hedgehog HCA (shHCA), and u-HCA. Of these, b^ex3^HCA and shHCA are associated with malignant transformation and the risk of bleeding, respectively. Although no genetic analysis was performed in the present case, Nault et al. reported that approximately 50% of u-HCA cases per the WHO classification would be classified into a new subgroup. Of the cases of u-HCA based on the WHO classification, those with hemorrhage were found to be classified as shHCA via genetic analysis, and the rest of the u-HCA cases were considered to have no risk of bleeding or malignant transformation. In the future, the nature of u-HCA will hopefully be clarified on the basis of such genetic analysis results. Consequently, clearer guidelines in diagnosis and treatment selection may emerge, and further studies on HCA are expected.^11,12^

There have been more than 150 reports of liver tumors with pedicled growth in the last 50 years. However, there are many cases of hemangiomas and HCC, and fewer than 10 cases of hepatocellular adenomas. The reported cases of liver tumors with pedicled growth were published before WHO classification, and details such as type are unknown. The present case is considered to be a rare case that could be diagnosed as liver-derived by blood flow evaluation by CT.

## Conclusions

We report a case of a large pedunculated HCA in the extrahepatic pelvic cavity. This case was challenging to diagnose because of an abnormally high PIVKA-II level and it was resected laparoscopically.

The authors declare no conflicts of interest associated with this manuscript.

Written informed consent was obtained from the study participants, including consent to participate and to publish the findings.

## Figures and Tables

**Table1:** Blood biochemistry at hospital admission

WBC	9900 /μl	ALP	241 U/l
Hb	45 g/dl	LAP	85 U/l
Plt	380 10^3^/μl	**γ-GTP**	**114 U/l**
PT	99 %	CHE	358 U/l
PT (INR)	1.01	Na	141 mEq/l
APTT	35.1 s	K	4.2 mEq/l
CRP	0.10 mg/dl	Cl	103 mEq/l
TP	7.6 g/dl	BUN	19 mg/dl
Alb	4.2 g/dl	Creatinine	0.42 mg/dl
T-bil	0.6 mg/dl	T-chol	194 mg/dl
D-bil	0.3 mg/dl	HBs Ag	(–)
AST	20 U/l	HBs Ab	<8
ALT	7 U/l	HBc Ab	(–)
LDH	179 U/l	HCV Ab	(–)
		**PIVKA-II**	**1386 mAU/ml**
		AFP	2.4 ng/ml

γ-GTP and PIVKA II levels were high, but there were no other abnormalities. WBC: white blood cell, Hb: hemoglobin, Plt: platelet, PT: Prothrombin time, INR: International Normalized Ratio, APTT: activated partial thromboplastin time, CRP: C-reactive protein, TP: total protein, Alb: albumin, T-Bil: total bilirubin, D-bil: direct bilirubin, AST: aspartate aminotransferase, ALT: alanine aminotransferase, LDH: lactase dehydrogenase, ALP: alkaline phosphatase, LAP: leucine aminopeptidase, γ-GTP: gamma-glutamyl transpeptidase, CHE: cholinesterase, BUN: blood urea nitrogen, T-chol: total cholesterol, HBs-Ag: hepatitis B surface antigen, HBs-Ab: hepatitis B surface antibody, HBc-Ag: hepatitis B core antigen, HCV-Ab: hepatitis C antibody, AFP: α-fetoprotein, PIVKA II: protein induced by vitamin K absence/antagonist-II
